# Supplementary material for: The Impact of Acute Ingestion of a Ketone Monoester Drink on LPS-Stimulated NLRP3 Activation in Humans with Obesity
Source: Nutrients. 2020 Mar 23;12(3):854. doi: 10.3390/nu12030854 (PMC7146505; doi:10.3390/nu12030854)
Supplement: Supplementary file 1 [file nutrients-12-00854-s001.pdf]

Supplementary Materials

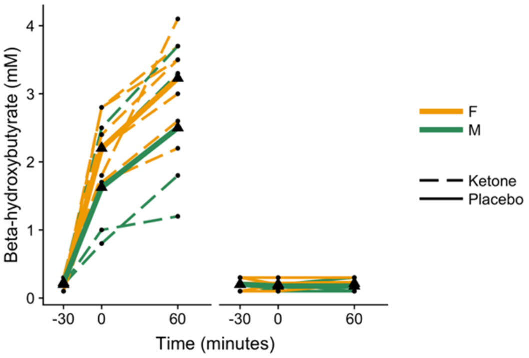

Supplemental Figure 1. Blood beta-hydroxybutyrate (β-OHB) following ketone or placebo drink ingestion disaggregated by sex. Individual participant data are shown with the ketone condition represented by dashed lines and the placebo condition represented by solid lines. Means are shown by bolded lines with triangles. Females are shown in blue (N = 7), and males are shown in red (N = 4). N = 11.

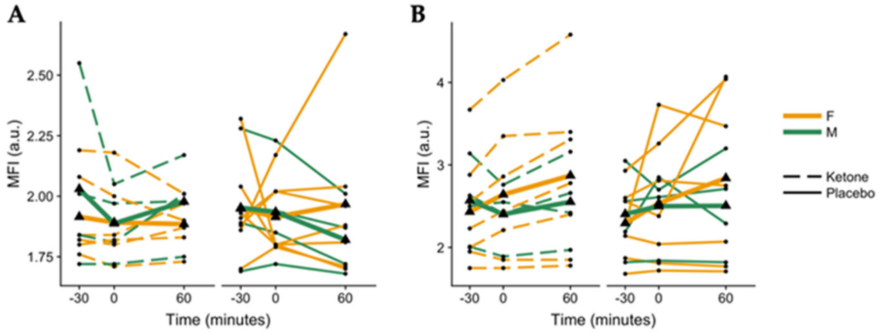

Supplemental Figure 2. Median fluorescence intensity (MFI) representative of active caspase-1 in human monocytes as quantified by flow cytometry disaggregated by sex. **A)** No changes were observed in unstimulated human monocytes. **B)** LPS-stimulated human monocytes. Individual participant data are shown with the ketone condition represented by dashed lines and the placebo condition represented by solid lines. Means are shown by bolded lines with triangles. Females are shown in yellow (N = 7), and males are shown in green (N = 4). N = 11.

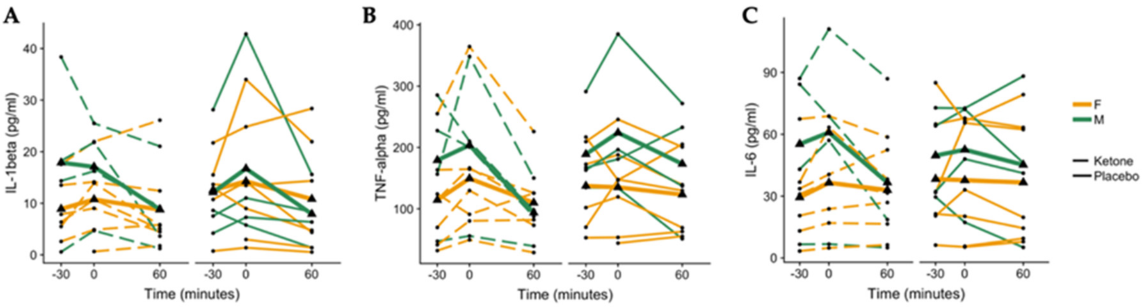

Supplemental Figure 3. Secreted cytokines from LPS-stimulated whole blood cultures grouped by sex. **a)** Secreted interleukin (IL)-1beta. **b)** Secreted interleukin (IL)-6. **c)** Secreted tumor necrosis factor (TNF)-alpha. Individual participant data are shown with the ketone condition represented by dashed lines and the placebo condition represented by solid lines. Means are shown by bolded lines with triangles. Females are shown in blue (N = 7), and males are shown in red (N = 4). N = 11.

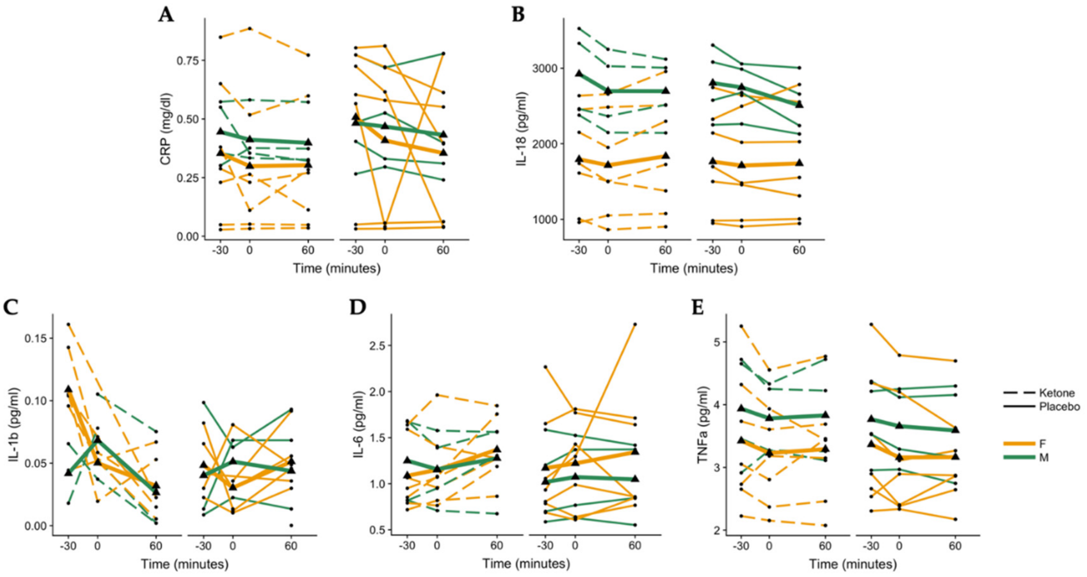

Supplemental Figure 4. Inflammatory markers in plasma at disaggregated by sex. A) C-reactive protein (CRP); B) interleukin 18 (IL-18); C) interleukin 1 beta (Il-1β). A total of ten samples were below the detection limit and three outliers were excluded; D) interleukin 6 (IL-6); E) tumour necrosis factor alpha (TNFα). Individual participant data are shown with the ketone condition represented by dashed lines and the placebo condition represented by solid lines. Means are shown by bolded lines with triangles. Females are shown in yellow (*N* = 7), and males are shown in green (*N* = 4). *N* = 11.

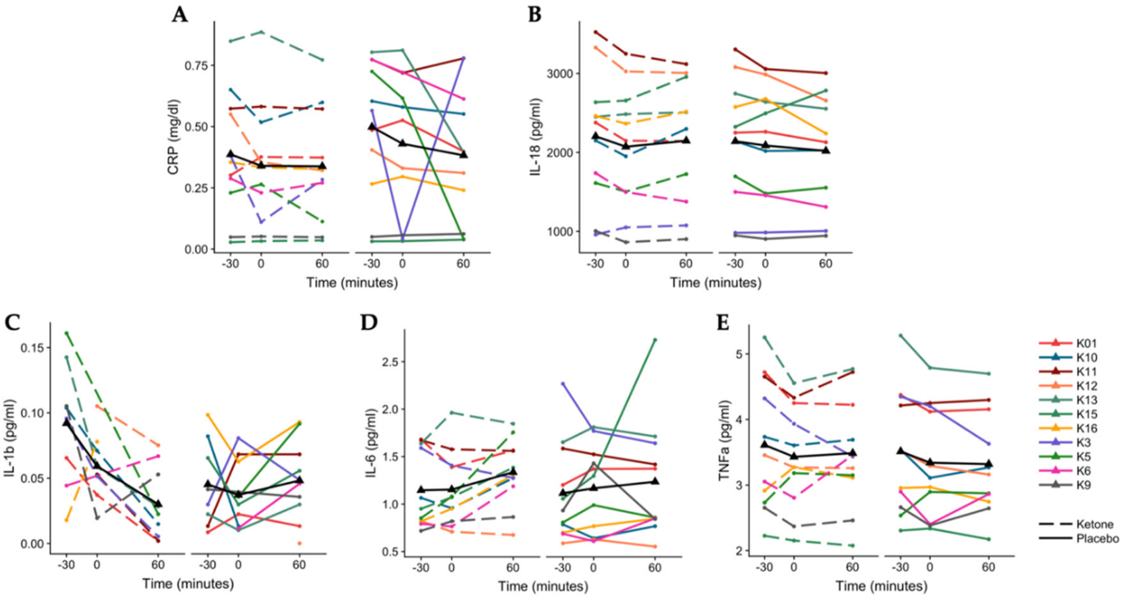

Supplemental Figure 5. Inflammatory markers in plasma with individual participants colour coded. A) C-reactive protein (CRP); B) interleukin 18 (IL-18); C) interleukin 1 beta (Il-1β). A total of ten samples were under the detection limit; D) interleukin 6 (IL-6); E) tumor necrosis factor alpha (TNFα). Colored lines are representative of individual participant data and are matched between conditions and plots. Mean is represented by the black line with triangles. Dashed lines are representative of the ketone condition, and solid lines are representative of the placebo condition. *N* = 11.
